# Disruptive behavior in the operating room: culture, responsibility, and the role of anesthesiologists

**DOI:** 10.1186/s44158-025-00261-y

**Published:** 2025-07-01

**Authors:** Michele Introna, Elena Giovanna Bignami

**Affiliations:** 1 Neurointensive Care Unit, Department of Neurosurgery, Fondazione IRCCS Istituto Neurologico Carlo Besta, Milan, Italy; 2 Department of Anesthesiology, University of Groningen, University Medical Center Groningen, Groningen, The Netherlands; 3 Anesthesiology, Critical Care and Pain Medicine Division, Department of Medicine and Surgery, University of Parma, Parma, Italy

**Keywords:** Equality, Anesthesiology, Surgery, Leadership

Dear Editor,

We write in response to the widely circulated video of a surgical incident at the Policlinico Tor Vergata, in which a senior surgeon verbally assaulted a colleague during an operation. The episode, captured on video and disseminated through national media, has sparked public outrage and institutional concern.

The surgeon involved later defended his actions with the following words:

“The operating room is a frontline where we fight, quite literally, between life and death. In those moments, every second counts. The responsibility is mine, entirely mine. And if I perceive a concrete risk to the patient, it is my duty to act. Our legal system calls this “a state of necessity,” and it is a full justification.”

While this justification may appear to appeal to the ethos of responsibility, it reveals a deeper and more troubling cultural issue within certain segments of medical practice. In fact, we believe the excuse is more damaging than the act itself.

The claim of “total” responsibility by the surgeon reflects problematic individualistic interpretation of clinical accountability. Modern medicine is a multidisciplinary endeavor, particularly in the operating room, where anesthesiologists, nurses, and surgical assistants share accountability. To deny this shared responsibility not only misrepresents reality, but also fuels a hierarchical culture that is incompatible with patient safety.

The surgeon further stated:

“We live in a world that often seems upside-down, where people superficially judge what they do not understand. Yet there are places, like the operating room, where there is no room for superficiality or approximation.”

As anesthesiologists, we know and understand this world well. And we also know how this very rhetoric has historically been used to excuse abuse, silence dissent, and perpetuate unjust power dynamics [1, 2].

Lastly, he claimed: “If I were on that table, I’d want the surgeon to do everything possible to keep me alive. Even yelling, if necessary. I wouldn’t want someone who stays silent out of fear of media backlash or political correctness. My job is not a performance. It’s science, technique, instinct, cold blood—and, above all, responsibility.”

As the surgeon involved is listed among the editorial board members of the *British Journal of Surgery*, he is surely familiar with the extensive literature from the past 30 years showing how such behaviour increases the risk of medical error. It promotes a culture of fear and silence, discourages open communication, and undermines the safety of the entire operating team [3]. In the modern surgical environment, professionalism, emotional regulation, and team-based accountability are not optional—they are essential for safe, effective care. Romanticizing aggression as a form of commitment or courage is not only scientifically unfounded, it is ethically indefensible.

It is also the responsibility of the anesthesiology community—and of the medical community at large—to exert pressure for systemic change, especially in countries like Italy, where workplace abuse and gender disparities are still too often tolerated. This tolerance is evident in the professor’s own words of justification, which reflect a culture that continues to excuse unacceptable behavior under the guise of professional duty. It falls upon the healthcare professional workforce to lower the threshold of tolerance toward such behaviors, with the clear objective of creating healthier work environments and ensuring greater safety for our patients.

## Data Availability

No datasets were generated or analysed during the current study.
